# Supplementary material for: Coupling spatial segregation with synthetic circuits to control bacterial survival
Source: Mol Syst Biol. 2016 Feb 29;12(2):859. doi: 10.15252/msb.20156567 (PMC4770385; doi:10.15252/msb.20156567)
Supplement: Supplementary file 1 — Appendix [file MSB-12-859-s001.docx]

*Appendix* **to**

# Coupling spatial segregation with synthetic circuits to control bacterial survival

Shuqiang Huang^a,1^, Anna Jisu Lee^a,1^, Ryan Tsoi^1^, Feilun Wu^1^, Ying Zhang^3^, Kam W Leong^3^ and Lingchong You^1,2^*

^1^ Department of Biomedical Engineering, Duke University, Durham, NC, USA

^2^ Center for Genomic and Computational Biology, Duke University, Durham, NC, USA

^3^ Department of Biomedical Engineering, Columbia University, New York, NY, USA

^a^ Equal contributions to this work

* Correspondence should be addressed to L.Y. ([you@duke.edu](mailto:you@duke.edu))

**Contents**

**Appendix Table S1.** Non-dimensionalized model parameters………………………………………...........3

**Appendix Figure S1.** Mechanisms of three self-addiction circuits for density-dependent survival..……..…4

**Appendix Figure S2.** Evaluation on the density dependency of *E. coli* MC4100 not carrying the BlaM circuit………………………………………………………………………………………………………5

**Appendix Figure S3.** Fabrication of alginate-PLL-alginate (APA) microcapsules……………………...…..6

**Appendix Figure S4.** Microbial swarmbot capsule………….……………………………………………..7

**Appendix Figure S5.** Simulated density-dependent survival of engineered bacteria in microbial swarmbot under a static condition…………...……………………………………………………………………..….8

**Appendix Figure S6**. A typical set of simulated time courses from the two-compartment model at the static growth condition……………………………………………………………………………................9

**Appendix Figure S7.** Modulate safeguard by increasing the volume ratio (V_R_)……………………...…….10

**Appendix Figure S8.** Measurement of cell density in the flow-out medium........…………………………11

**Appendix Figure S9.** Replicate experiments of Figure 3E and Figure 3F………........……………………12

**Appendix Figure S10.** Replicate experiments of safeguard modulation by controlling nutrient and antibiotic concentrations, under a pulsing condition……………………………..........…………………....13

**Appendix Figure S11.** Quantification of safeguard control with QS-CAT and QS-BlaM circuits……...….14

**Appendix Figure S12.** Patchy growth on the PDMS surface at the pulsing condition and in absence of antibiotics…………………………………………………………………………………………………15

**Appendix Figure S13.** QS-BlaM circuit functionality with QS regulation .……….……………….......…..16

**References**……………………………………………………………………………….……....……….17

| **Variables** | **Definition** | **Description** | **Base value** |
| --- | --- | --- | --- |
| *n* | $\frac{N}{N_{m}}$ | Cell density | variable |
| *s* | $\frac{S}{K_{s}}$ | Nutrient concentration | variable |
| *a* | $\frac{A}{K_{amp}}$ | Antibiotic concentration | variable |
| *b* | $\frac{v_{max}B}{\mu_{max}K_{amp}}$ | BlaM concentration | 0 |
| *g* | $\frac{G}{\mu_{max}},$ | Growth | n/a |
| *l* | $\frac{L}{d_{A}}$ | Lysis | n/a |
| $\tau$ | $t \mu_{max}$ | Time | n/a |
| $\gamma_{1}$ | $\left( \frac{d_{A}}{\mu_{max}} \right)$ | Death rate of cells | 2.0 |
| $\gamma_{2}$ | $\left( \frac{{\alpha N}_{m}}{K_{S}} \right)$ | Rate of nutrient consumption | 0.5 |
| $\gamma_{3}$ | $\left( \frac{d_{B}}{\mu} \right)$ | Rate of intrinsic BlaM degradation | 0.2 |
| $\beta_{1}$ | $\left( \frac{k_{r}N_{m}}{K_{s}} \right)$ | Percent recovery of nutrients upon cell lysis | 0.8 |
| $\beta_{2}$ | $\left( \frac{v_{max}N_{m}k_{bla}}{\mu_{max}K_{amp}} \right)$ | Synthesis and release rates of BlaM | 5 |
| *σ_1_* | $\frac{K_{lysis}}{K_{amp}}$ | Half-maximal constant for cell lysis by antibiotics | 0.2 |
| $V_{R}$ | n/a | Capsule to chamber volume ratio | 0.1 |
| $g_{0}$ | $\frac{G_{0}}{\mu_{max}}$ | Minimum threshold of growth rate for lysis | 0.05 |
| $f_{n}$ | n/a | Transport rate of cells across APA shell | 0.0002 |
| $f_{s}$ | n/a | Transport rate of nutrient molecules across APA shell | 0.6 |
| $f_{a}$ | n/a | Transport rate of antibiotics across APA shell | 0.6 |
| $f_{b}$ | n/a | Transport rate of BlaM across APA shell | 0.1 |

**Table S1. Non-dimensionalized model parameters**. Using parameter values from previous literatures as reference (Hou & Poole, 1969; Osuna et al, 1995; S. C. Schultz, 1987; Schultz Sc, 1987; Zlokarnik et al, 1998), the non-dimensionalized model parameters were our educated evaluation on experimentally relevant numbers.


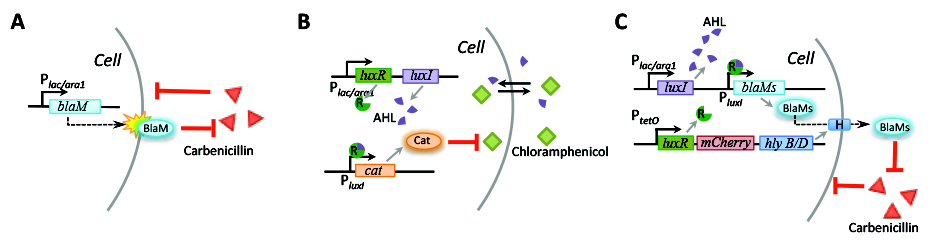


**Figure S1. Mechanisms of three self-addiction circuits for density-dependent survival.**

1. **The BlaM circuit** consists of constitutive expression of BlaM. Upon treatment by carbenicillin, BlaM is released from lysed cells into the culture where it can degrade the antibiotic. If the initial cell density is sufficiently high, the released BlaM can degrade the antibiotic sufficiently fast to enable population survival.
2. **The QS-CAT circuit** couples production of CAT to QS. CAT is expressed as AHL accumulates at a high density, enabling resistance to chloramphenicol.
3. **The QS-BlaM circuit** couples production of BlaM to QS. The main difference of this gene circuit from the BlaM circuit is that the production of BlaM is activated by QS. BlaM is produced as AHL accumulates at a high density. The protein is tagged with a HlyAs sequence and can be exported by the HlyB/D transport apparatus. It can also be released due to lysis of a subpopulation of cells upon antibiotic treatment. Sufficient accumulation of BlaM can enable population survival at a given antibiotic dose.

*E. coli* MC4100 + pZS31GFP

High density [Amp]=100

**Figure S2. Evaluation on the density dependency of *E. coli* MC4100 not carrying the BlaM circuit.**

1. **Growth dynamics of MC4100 cells in liquid culture.** *E. coli* MC4100 strain not carrying BlaM circuit was treated with varying carbenicillin concentrations to confirm that they do not intrinsically exhibit density-dependency. The cells were inoculated in supplemented M9 medium in 96-well plates at varying initial cell densities and cultured for 15 hours at 37°C to measure the final cell density. As shown in this result, the cells did not exhibit significant density-dependent growth upon treatments with either 30 or 100 μg/mL carbenicillin. Without carbenicillin treatment, the cells could fully reach its carrying capacity as indicated by the black line ([Cb] = 0 μg/mL).
2. **Growth dynamics of MC4100 cells expressing only GFP in the microbial swarmbot.** An overnight culture of MC4100 cells constitutively expressing GFP but lacking the BlaM circuit was encapsulated in swarmbot capsules at a high initial cell density, as in Figure 3H. The resulting swarmbot capsules were then incubated at 37°C for 8 hours as described in **Materials and Methods**. After pre-incubation the swarmbot was cultured in an M9 medium containing 100 μg/ml carbenicillin. These cells were killed at a fast rate, and could not maintain population-level survival. The solid line indicates the growth in the swarmbot while the dotted line indicates that in the culturing chamber.

**Figure S3. Fabrication of alginate-PLL-alginate (APA) microcapsules.**

**(A-D) Fabricating procedures of an SU8 mold for a PDMS template.** Soft lithography was employed to make a photoresist mold that involved photoresist coating, soft bake, UV exposure, post bake and development.

**(E-H) Production of alginate particles transferred from the PDMS template.** Mixed PDMS polymer was poured on the mold to replicate the pattern. An alginate solution containing engineered bacteria was then poured on the PDMS template. After being solidified by the calcium ion, the alginate particles were detached from the template by sonication.

**(I-L) Generation of APA microcapsules with a hollow structure.** Once collected, the alginate particles were coated by poly (L-lysine) and by alginate solution sequentially, and finally liquefied by sodium citrate to generate the hollow structure.


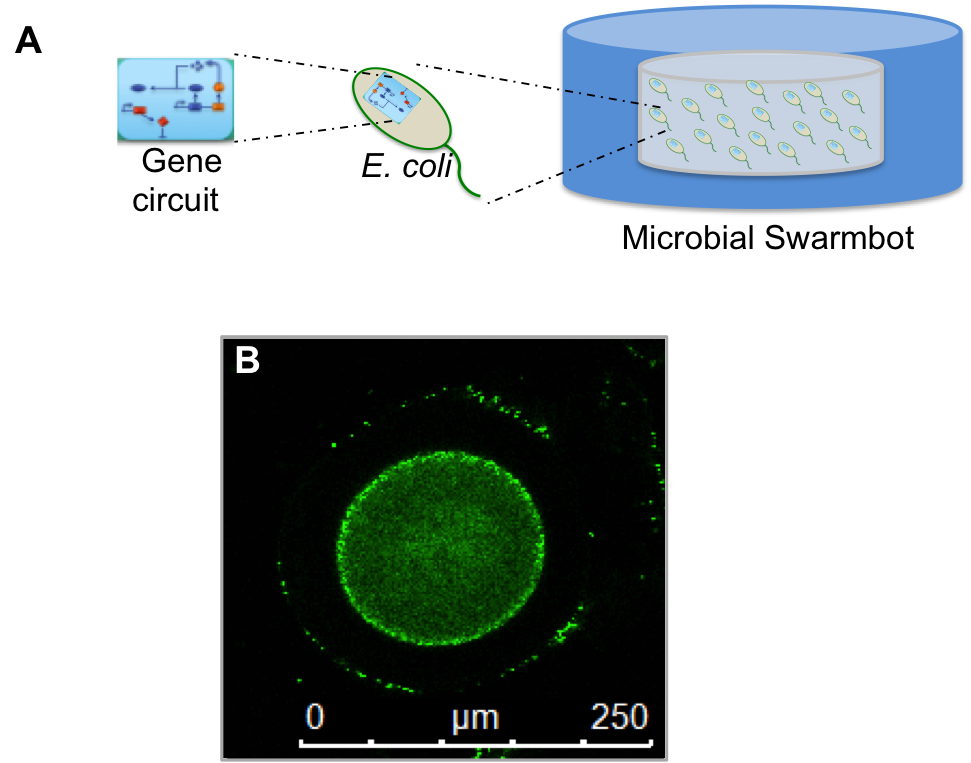


**B**


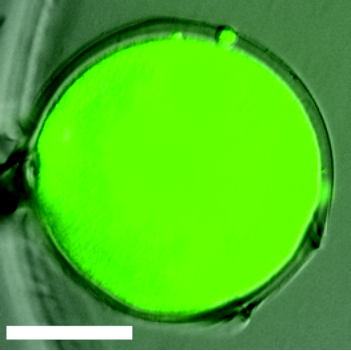


**Figure S4.** **Microbial swarmbot capsule.**

1. **Schematic of a cylindrical swarmbot capsule.** Each swarmbot contains engineered bacteria carrying specific gene circuits.
2. **Scanning image of a swarmbot capsule.** The diameter of swarmbot is ~250 µm and shell thickness ~15 µm. The green fluorescence inside the swarmbot capsule is from the engineered *E. coli*. The scale bar represents 100 µm.

Initial density in swarmbot

Final density in swarmbot

*a* = 0

*a* = 0.2

*a* = 0.8

**Figure S5. Simulated density-dependent survival of engineered bacteria in microbial swarmbot under a static condition.** The bacteria exhibit increased threshold of initial density for higher *a*. Each line represents different levels of antibiotic treatment.


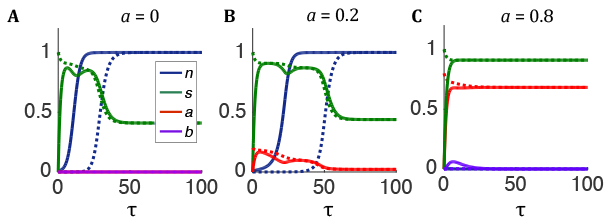


**Figure S6**. **A typical set of simulated time courses from the two-compartment model at the static growth condition.** The blue, green, red, and purple curves represent each component as shown in the legend. The solid lines correspond to the dynamics in the swarmbot, while the dashed lines indicate those in the chamber.

1. The cells can quickly initiate growth where *a* = 0. Concentration of nutrients *s* decreases as they are consumed from cell growth. Concentrations of *a* and *b* remains at 0 because there is no *a* and corresponding triggered release of *b.*
2. After longer initiation time for growth, the cells can reach the full carrying capacity where *a* = 0.2, which is the half-maximal threshold value for the antibiotic killing. At *a* = 0.2, initially, the populations take some time to degrade Cb by BlaM released from subpopulation death. As *a* < 0.2, the populations are able to initiate growth roughly around τ = 50.
3. At *a* = 0.8, the cells cannot produce sufficient amount of BlaM. Thus, even when all cells in the population die, the released BlaM fails to degrade *a* below the threshold level for survival.


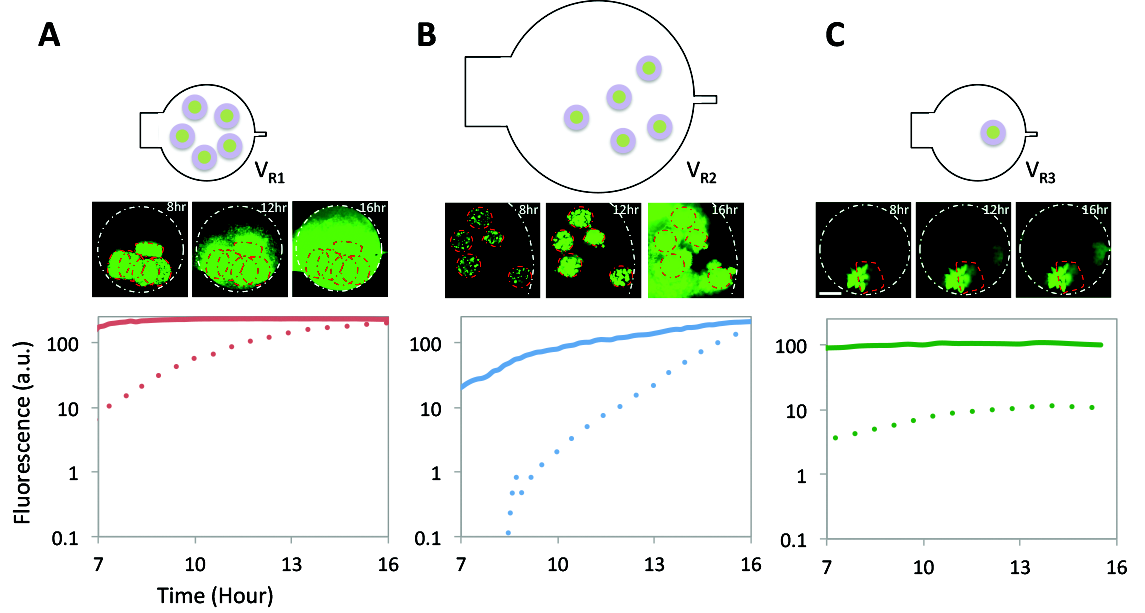


**Figure S7. Modulate safeguard by increasing the volume ratio (V_R_).** (A) 1mm (in diameter) chamber contained 5 swarmbot capsules (V_R1_), while (B) 2mm chamber contained 5 capsules (V_R2_), and (C) 1mm chamber contained only 1 capsule (V_R3_). The heights of two different chambers were the same, thus, V_R1_ < V_R2_ < V_R3_. The concentrations of Cb were all set as 30 µg/ml, and the white circles denote the location of culturing chamber while the red polygons the location of capsules. The quantitative curves further illustrated the increasing safeguard control by the increased V_R_ value. The solid lines indicated the averaged growth in the swarmbot capsules while the dotted lines the growth in the chambers.

**Figure S8. Measurement of cell density in the flow-out medium.** For the pulsing perfusion in Figure 5B, the flow-out medium of 0.04% glucose with 0 and 100 µg/ml carbenicillin were collected in the culturing tubes and also cultured at 37˚C. After 16 hours, cell density (OD600) was measured by using plate reader. Cell growth was detected for the perfusion of medium without the antibiotic (left), but not for the one with 100 µg/ml carbenicillin (right), which indicated no viable escaped bacteria outside swarmbot capsule. Error bars represented the standard deviation of triplicate measurements.

**Figure S9. Replicate experiments of Figure 3E and Figure 3F.** (A) swarmbots with 100 µg/ml Cb and (B) swarmbots with 0 µg/ml Cb. Green lines indicated the high initial cell density while the red lines the low initial density. The solid lines referred to the growth in the swarmbot capsules while the dashed lines the growth in the culturing chambers. The snapshot showed the growing status at 16^th^ hour.

**Figure S10. Replicate experiments of safeguard modulation by controlling nutrient and antibiotic concentrations, under a pulsing condition.**  The nutrient levels, antibiotic concentrations and fluidic settings were the same as Figure 5. The images represented the snapshots at 16^th^ hour.

**Figure S11. Quantification of safeguard control with QS-CAT and QS-BlaM circuits.** The experimental was the same sets as Figure 6. (A) Engineered system with QS-CAT, the swarmbots were cultured at a static condition in presence of either 0 or 100 µg/ml Cm. (B) QS-CAT systems were cultured at a pulsing condition. (C) QS-BlaM system at a static condition with either 0 or 100 µg/ml Cb. The quantification further validated the safeguard performance for different modular systems. The solid lines indicated the growth in the swarmbots while the dash lines depicted that in the chambers.

**Figure S12. Patchy growth on the PDMS surface at the pulsing condition and in absence of antibiotics.** (A-C) Bacteria with BlaM circuit and different nutrient levels exhibited the concentrated growth at different time due to the difference in growth rate. (D) Another replicate of bacteria with QS-CAT circuit showing patchy growth, similar as that shown in Figure 6. Red arrows indicate the locations of patchy growth, which was likely due to the heterogeneity of the PDMS surface.

**Figure S13. QS-BlaM circuit functionality with QS regulation.** (A) The cells carrying QS-BlaM circuit were cultured with varying initial densities without any inducer to activate the QS. Carbenicillin concentration in the samples were either 0 µg/mL (blue line, control) or 30µg/ml (red line). (B) QS module was fully induced with 0.5mM IPTG, 0.1% arabinose for induction of LuxI and with 100nM aTc for LuxR and Hly B/D transporter (Appendix Fig S1). Compared to the condition where cells were growing without any inducer in (A), the survival threshold was lowered by 2.2 fold for cells growing at 30µg/mL condition. Each error bar indicates the standard deviation of triplicate measurements.

**References**

Hou JP, Poole JW (1969) Kinetics and mechanism of degradation of Ampicillin in solution. *Journal of Pharmaceutical Sciences* **58:** 447-454

Osuna J, Viadiu H, Fink AL, Soberón X (1995) Substitution of Asp for Asn at Position 132 in the Active Site of TEM -Lactamase: ACTIVITY TOWARD DIFFERENT SUBSTRATES AND EFFECTS OF NEIGHBORING RESIDUES. *Journal of Biological Chemistry* **270:** 775-780

S. C. Schultz GD-M, J. J. Neitzel, J. H. Richards (1987) Stability of wild-type and mutant RTEM-1 beta-lactamases: effect of the disulfide bond. *Proteins* **2:** 290-297

Schultz Sc D-MGNJJRJH (1987) Stability of wild-type and mutant RTEM-1 beta-lactamases: effect of the disulfide bond. *Proteins* **2:** 290-297

Zlokarnik G, Negulescu PA, Knapp TE, Mere L, Burres N, Feng L, Whitney M, Roemer K, Tsien RY (1998) Quantitation of Transcription and Clonal Selection of Single Living Cells with β-Lactamase as Reporter. *Science* **279:** 84-88
