# Supplementary material for: Coupling spatial segregation with synthetic circuits to control bacterial survival
Source: Mol Syst Biol. 2016 Feb 29;12(2):859. doi: 10.15252/msb.20156567 (PMC4770385; doi:10.15252/msb.20156567)
Supplement: Supplementary file 2 — Movie EV1 [file MSB-12-859-s002.zip › Movie_EV1/Read_me-movie_EV1.rtf]

Movie  EV1: No safeguard of QS-BlaM in absence of carbenicillin. Related to Figure6
