# Supplementary material for: Coupling spatial segregation with synthetic circuits to control bacterial survival
Source: Mol Syst Biol. 2016 Feb 29;12(2):859. doi: 10.15252/msb.20156567 (PMC4770385; doi:10.15252/msb.20156567)
Supplement: Supplementary file 3 — Movie EV2 [file MSB-12-859-s003.zip › Movie_EV2/Read_me-movie_EV2.rtf]

Movie  EV2: Established safeguard of QS-BlaM in presence of 100µg ml-1 carbenicillin. Related to Figure6.
